# Structural Diversity of Silver Fluorinated β-Diketonates: Effect of the Terminal Substituent and Solvent

**DOI:** 10.3390/molecules27030677

**Published:** 2022-01-20

**Authors:** Evgeniia S. Vikulova, Taisiya S. Sukhikh, Sergey A. Gulyaev, Igor Yu. Ilyin, Natalia B. Morozova

**Affiliations:** 1 Nikolaev Institute of Inorganic Chemistry, Siberian Branch of Russian Academy of Sciences, 3, Academy Lavrentieva Avenue, 630090 Novosibirsk, Russia; sukhikh@niic.nsc.ru (T.S.S.); s.gulyaev@g.nsu.ru (S.A.G.); ilyin@niic.nsc.ru (I.Y.I.); mor@niic.nsc.ru (N.B.M.); 2 Department of Natural Sciences, Novosibirsk State University, 2 Pirogova Street, 630090 Novosibirsk, Russia

**Keywords:** silver, β-diketonate, fluorinated, coordination polymers, crystal structure, TGA

## Abstract

In order to demonstrate the role of the fluorination and some solvents in the structural organization of the Ag(I) coordination polymers with β-diketonate ligands (R^1^C(O)C_α_HC(O)R^2^)^−^ we synthesized a series of the compounds containing tfac- (R_1_ = CH_3_, R^2^ = CF_3_) and pfpac- (R_1_ = CH_3_, R^2^ = C_2_F_5_) anions. Solvent-free [Ag(L)]_∞_ (L = tfac **1**, pfpac **2**) compounds and the corresponding acetonitrile and toluene adducts have been characterized by elemental analysis and/or NMR, IR and single-crystal XRD. This series includes five new coordination polymers. Compound **1** is a 3D coordination framework based on Ag–O_chelate/bridge_, Ag–C_α_ bonds, and argentophilic interactions. An increase in the fluorinated group leads to a chain coordination polymer **2** of an unusual structural organization. These chains can be represented as a “DNA-type”, where two intertwined helices based on Ag–O_chelate_ and Ag–C_α_ bonds are connected through Ag–O_bridge_ ones. Two structural types of chain coordination polymers, [Ag(tfac)(CH_3_CN)] and [Ag_2_(L)_2_(solvent)], have been revealed for the adducts. The latter structural type differs significantly from the previously studied toluene and acetonitrile adducts of fluorinated Ag(I) β-diketonates of the same stoichiometry. Thermal analysis in helium showed that both **1** and **2** decompose to metallic silver with the compound of pfpac-ligand being slightly more stable.

## 1. Introduction

β-Diketonate ligands (R^1^C(O)C_α_RC(O)R^2^)^−^ are widely demanded building blocks in coordination chemistry, which are distinguished by a chelating effect and internal conjugation, providing stability, and their great structural versatility by their varying terminal groups (R^1^ and R^2^) and the substituent at the methine carbon atom (C_α_) [1,2,3,4]. This allows the forming of complexes of various structural organization and dimension. In particular, for silver, fluorinated β-diketonate ligands were used for constructing coordination polymers with macroligands [5,6] or exo-N-donor synthons [7,8], stabilizing the multinuclear ethynide or thiolate clusters [9,10,11], and obtaining polymer, di- or mononuclear precursors [12,13,14] for chemical vapor deposition processes.

However, much less attention has been paid to the scrutiny of pristine silver(I) fluorinated β-diketonates, although a rich structural diversity can also be expected there. In addition to the different modes of anion coordination, a feature of silver in such a ligand environment includes the Ag–C_α_ bonds with the methine carbon atom and argentophilic interactions.

**Scheme 1:**
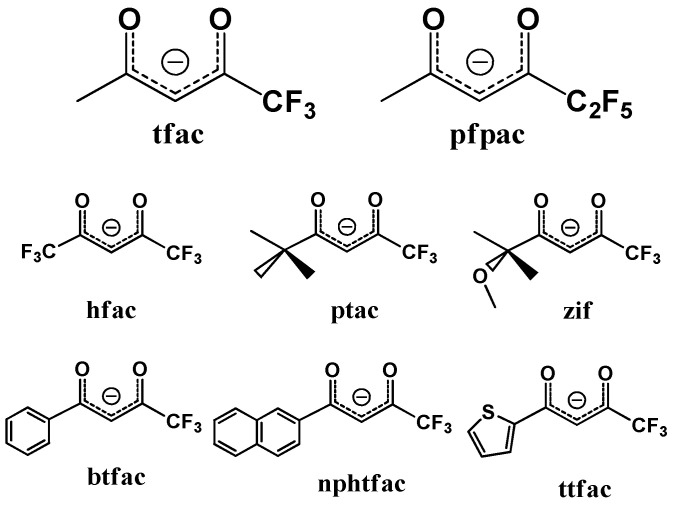
The β-diketonate ligands discussed in this work.

The realization of these possibilities is determined by the structure of the β-diketonate ligand. Several silver (I) β-diketonates have been structurally characterized, where only the influence of the non-fluorinated terminal group can be observed: R = H, R^1^ = CF_3_, R^2^ = CH_3_ [15], C(CH_3_)_3_ [16], C(OCH_3_)(CH_3_)_2_ [17], C_6_H_5_ [18] (Scheme 1). In particular, an increase in the substituent reduces the dimensionality of the polymer structure to 1D. These tapes are corrugated, and for the *tert*-butyl and phenyl substituents, they are organized in the same way, with a with significant kink of the chelate metallocycle [16,18], while an additional donor atom (O_CH3_), which is coordinated to silver, reduces the tightness of the structure [17]. The effects of changing the substituent at the methine atom or fluorinated group have not been studied.

Another interesting capacity to design the structure of silver(I) β-diketonates is the coordination of solvents of a different nature. For example, coordination polymers [Ag_2_(R^1^C(O)C_α_HC(O)R^2^)_2_Q]_∞_ are known for Q = water [19], toluene, tetrahydrofuran (THF) [20] (R^1^ = R^2^ = CF_3_), and acetonitrile [21] (R^1^ = CF_3_, R^2^ = C_6_H_5_, C_10_H_7_, C_4_H_3_S) (Scheme 1). Despite the same stoichiometry, the structures of these polymers differ in their dimensions (2D for water and toluene adducts, 1D for THF and acetonitrile adducts) and further in their β-diketonate coordination modes and silver environment.

In addition to the fundamental interest for the structural features and the development of an understanding of the silver coordination capabilities, Ag(I) β-diketonates and their adducts with solvents are also useful for practical applications. For example, some polymers have exhibited solid phase luminescence [21]. Due to their low thermal stability (<200 °C), these compounds can be used to obtain Ag-based coatings and nanoparticles by thermal or supercritical fluid deposition [15,22,23]. The decomposition temperature range and solubility of the target solvent can be tuned by a ligand structure modification. Moreover, photosensitivity of silver β-diketonates makes it possible to obtain functional materials even at room temperature [22,24]. In addition, pristine silver(I) coordination polymers are receiving attention as antimicrobial materials [25,26,27].

Summarizing the above, our work is devoted to revealing the effect of a fluorinated substituent (CF_3_ → C_2_F_5_) and a solvent (CH_3_CN, toluene) on the structural and thermal properties of Ag(I) β-diketonates (Scheme 1). All the complexes with 1,1,1,2,2-pentafluoro-3,5-hexanedione (Hpfpac, C_2_F_5_C(O)CH_2_C(O)CH_3_) have been synthesized for the first time. For the counterpart, 1,1,1-trifluoro-2,4-pentanedione (Htfac, CF_3_C(O)CH_2_C(O)CH_3_), some information on the silver complex has been already presented [15], however, without any data on the discussed adducts. In addition, despite the description of the [Ag(tfac)]_∞_ structure [15], no corresponding data are available in the Cambridge Structural Database. Thus, here we present the synthesis and characterization of the Ag(I) fluorinated β-diketonate coordination polymers and their solvent adducts, the crystal structures of [Ag(L)]_∞_ (L = tfac **1**, pfpac **2**), [Ag(tfac)(CH_3_CN)]_∞_ **1a**, [Ag_2_(pfpac)_2_(CH_3_CN)]_∞_ **2a**, [Ag_2_(L)_2_(C_7_H_8_)]_∞_ (L = tfac **1t**, pfpac **2t**), and the comparative thermogravimetric study. The features of the new structures versus the related coordination polymers [20,21] will be also discussed.

## 2. Materials and Methods

### 2.1. Sourses and Analitical Methods

All reagents and solvents were commercially available. For synthesis under ambient conditions no additional purification was performed. For synthesis under the inert conditions (Schlenk technique), all the liquids were previously dehydrated and then degassed by distillation in argon.

Elemental analyses were performed with a Carlo Erba Model 1106 instrument (for C, H, N) or by a standard spectrophotometric method with La complex of alizarin complexone (for F) [28]. The errors of the measurements did not exceed 0.5 abs. wt.%.

IR (infrared) spectra were recorded with a Scimitar FTS 2000 spectrometer in the wavenumber range of 4000–400 cm^–1^. The samples were pellets with KBr or fluorinated oil.

NMR (nuclear magnetic resonance) spectra were recorded on a Bruker Avance 500 Plus spectrometer (^1^H: 500.129 MHz, 13C: 125.757 MHz) at 25 °C. The samples were dissolved in deuterated dimethylsulfoxide (DMSO-*d*_6_). Chemical shifts (δ, ppm) were calibrated with residual DMSO as the internal standard (^1^H = 2.50; ^13^C = 39.52) [29].

The thermogravimetry (TG) study was carried out with a Netzsch TG 209 F1 Iris thermobalance in a helium flow (30 mL min^−1^). Sample masses were 10 ± 1 mg; the heating rate was 10 °C∙min^−1^. The thermal effects were recorded with c-DTA^®^ software (Netzsch) for the calculated signal of differential thermal analysis (c-DTA). The final decomposition products were studied by X-ray powder diffraction (XRPD), with a Shimadzu XRD-7000 diffractometer (Tokyo, Japan) using CuKα radiation (Ni filter, OneSight linear detector). The steps of 2Θ were 0.0143° with an accumulation of 5 sec at a point in the range 10–65°.

### 2.2. Synthesis of [Ag(tfac)]_∞_ ***1***

The synthesis was carried out in air. 0.222 g of Ag_2_O (0.96 mmol) was placed in aluminum foil-wrapped flask, and then 35 mL of Et_2_O was added. The flask was then placed in water/ice bath for cooling. 0.4 mL of Htfac (2.10 mmol) was added dropwise with stirring. Synthesis continued for 2 h. White precipitate that appeared was filtered and dried in vacuum. The product was Ag_2_(tfac)_2_(H_2_O) **1w**. Yield: 90% (0.468 g, 0.87 mmol). IR spectrum (KBr, cm^−1^): 3430, 3266 (ν(O–H)), 3011, 2929, 2862 (ν(C–H)), 1655, 1621, 1552 (ν(C=O)), 1528 (ν(C=C) + δ(C–H)), 1512 (ν(C=O) + ν(C=C)), 1481 (ν(C=O)), 1438 (δ(CH_3_)), 1360 (ν(C=O)), 1282 (ν(C=C) + ν(C–CF_3_)), 1215 (δ(CH)), 1190 (ν(C–F)), 1132 (ν(C–C)), 1119 (δ(CF_3_)), 1021, 1000 (ρ(CH_3_)), 929, 847 (ν(C–CF_3_)), 808–800 (ν(C–C) + δ(C–C) + ν(C–CH_3_) + π(C–H)), 758 (ν(Ag–O) + ν(C–CH_3_) + chelate def.), 725 (ν(Ag-C) + ν(C–CH_3_) + δ(C=O)), 604 (ν(Ag–O)), 551 (ν(Ag–C)), 516 (ν(Ag–C)).

The slow ether extraction of **1w** in an evacuated sealed two-knee ampoule [30] leads to the crystals of the anhydrous [Ag(tfac)]_∞_ **1,** suitable for single-crystal X-ray diffraction (XRD) in the lower knee. The partial decomposition of **1w** was also observed in the upper knee. Anal. Calcd. (wt. %): for Ag_2_C_10_H_10_O_5_F_6_ **1**: C, 23.0; H, 1.5; F, 21.8. Found: C, 22.9; H, 1.8; F, 22.0. IR spectrum (KBr, cm^−1^): 2923 (ν(C–H)), 1635 (ν(C=O), 1532 (ν(C=C) + δ(C–H)), 1463 (ν(C=O)), 1360 (ν(C=O)), 1281 (ν(C=C) + ν(C–CF_3_)), 1203 (δ(CH)), 1189 (ν(C–F)), 1137 (ν(C–C)), 1117 (δ(CF_3_)),1020, 988 (ρ(CH_3_)), 919, 844 (ν(C–CF_3_)), 816 (ν(C–C) + δ(C–C) + ν(C–CH_3_)), 803 (π(C–H)), 757 (ν(Ag–O) + ν(C–CH_3_) + chelate def.), 724 (ν(Ag–C) + ν(C–CH_3_) + δ(C=O)), 603 (ν(Ag–O)), 555 (ν(Ag–C)), 517 (ν(Ag–C)).

### 2.3. Synthesis of [Ag(pfpac)]_∞_ ***2***

The synthesis in air was performed similarly for **1w**; however, the solvent volume was reduced to prevent product dissolution. Namely, for 0.190 g of Ag_2_O (0.82 mmol) we used 6.5 mL of Et_2_O and 0.3 mL of Hpfpac (1.98 mmol). Resulting white precipitate was filtered and dried in vacuum. The product was also agua-β-diketonate Ag_2_(pfpac)_2_(H_2_O) **2w**. Yield: 82% (0.430 g, 0.67 mmol). Anal. Calcd. (wt. %): for Ag_2_C_12_H_10_O_5_F_10_ **2w**: C, 22.5; H, 1.6; F, 29.7. Found: C, 22.9; H, 1.1; F, 30.3. IR spectrum (KBr, cm^−1^): 3430, 3272 (ν(O–H)), 2980, 2933 (ν(C–H)), 1645 (ν(C=O), 1518 (ν(C=C) + δ(C–H) + ν(C=O)), 1459 (ν(C=O)), 1360 (ν(C=O)), 1328 (ν(C–CF_3_)), 1214 (δ(CH)), 1192 (ν(C–F)), 1162 (ν(C–C)), 1123 (δ(CF_3_)), 1027, 992 (ρ(CH_3_)), 932 (ν(C–CF_3_)), 819 (ν(C–C) + δ(C–C) + ν(C–CH_3_)), 805 (π(C–H)), 757 (ν(Ag–O) + ν(C–CH_3_) + chelate def.), 726 (ν(Ag–C) + ν(C–CH_3_) + δ(C=O)), 620 (ν(Ag–O)), 561 (ν(Ag–C)), 531 (ν(Ag–C)), 496.

To obtain the anhydrous compound, we carried out a similar synthesis in a Schlenk apparatus using molecular sieves. The volume of ether here was such as to ensure complete dissolution of the target product (e.g., 30 mL for 56 mg of Ag_2_O). After 18 h stirring at room temperature, the reaction mixture was filtered using kieselguhr. A white powder [Ag(pfpac)]_∞_ **2** was obtained from a colorless mother liquor by evaporation of ether in vacuum (10^−2^ Torr). ^1^H NMR spectrum (DMSO-*d*_6_, 2.50 ppm): δ 5.02 (br s, 1H, C_α_–H), 1.96 (s, 3H, –CH3). ^13^C NMR spectrum (DMSO-*d*_6_, 39.52 ppm): 194.78 (s, C=O, pfpac), 169.00 (t, *J*_C,F_ = 20.7 Hz, C=O, pfpac), 118.97 (qt, *J*_1_ = 286.4, *J*_2_ = 37.0, –CF_3_, pfpac), 108.80 (tq, *J*_1_ = 264.0, *J*_2_ = 35.7, >CF_2_, pfpac), 91.79 (s, C_α_–H, pfpac), 29.95 (s, –CH_3_, pfpac).

Crystals of the anhydrous compound **2** suitable for single-crystal XRD were also grown by slow ether extraction of **2w** in an evacuated sealed two-knee ampoule. As for the tfac-based complex, the partial decomposition of the initial **2w** was observed in the upper knee.

### 2.4. Synthesis of Ag(I) β-Diketonate Adducts

Adducts with acetonitrile and toluene were obtained by recrystallization of Ag(I) aqua-β-diketonates **1w** and **2w** from the corresponding solvents under ambient conditions. The resulting solution was filtered to remove a small amount of brown precipitate, and then the solvent was evaporated in vacuum (10^−1^–10^−2^ Torr). The products were formed as white powders and corresponded to stoichiometry [Ag_2_(L)_2_Q]_∞_ (Q = CH_3_CN, toluene; L = tfac, pfpac). The yields were 90–95% for acetonitrile and 80% for toluene.

Ag_2_(tfac)_2_(CH_3_CN), ^1^H NMR spectrum (DMSO-*d*_6_, 2.50 ppm): δ 5.14 (br s, 1H, C_α_–H), 2.07 (s, (3H) × 0.5, –CH_3_, CH_3_CN), 1.98 (s, 3H, –CH_3_, tfac).

[Ag_2_(tfac)_2_(C_7_H_8_)]_∞_ **1t**, ^1^H NMR spectrum (DMSO-*d*_6_, 2.50 ppm): δ 7.25 and 7.18 (both m, (5H) × 0.5, C(Ph)-H, toluene), 5.05 (br s, 1H, C_α_–H), 2.30 (s, (3H) × 0.5, –CH_3_, toluene), 2.01 (s, 3H, –CH_3_, tfac).

[Ag_2_(pfpac)_2_(CH_3_CN)]_∞_ **2a**, ^1^H NMR spectrum (DMSO-*d*_6_, 2.50 ppm): δ 5.06 (br s, 1H, C_α_–H), 2.07 (s, (3H) × 0.5, –CH_3_, CH_3_CN), 2.02 (s, 3H, –CH_3_, pfpac).

[Ag_2_(pfpac)_2_(C_7_H_8_)]_∞_ **2t**, ^1^H NMR spectrum (DMSO-*d*_6_, 2.50 ppm): δ 7.25 and 7.18 (both m, (5H) × 0.5, C(Ph)-H, toluene), 5.13 (br s, 1H, C_α_–H), 2.30 (s, (3H) × 0.5, –CH_3_, toluene), 2.00 (s, 3H, –CH_3_, pfpac).

The crystals of the adducts suitable for single-crystal XRD were obtained by slow evaporation of the corresponding solvents at 5 °C. For all adducts, the stoichiometry of the crystals corresponded to powder samples, except with [Ag(tfac)(CH_3_CN)]_∞_ **1a**.

### 2.5. Single-Crystal XRD Study

Single-crystal XRD data for compounds **1**, **1a** and **1t** (Table S1, Supplementary Materials) were collected by a Bruker D8 Venture diffractometer with a CMOS PHOTON III detector and IμS 3.0 microfocus source (collimating Montel mirrors). For compounds **2**, **2a** and **2t** (Table S1, Supplementary Materials), the data collection was performed on a Bruker Apex DUO diffractometer equipped with a 4K CCD area detector using the graphite-monochromated radiation. All the experiments were carried at 150 K using MoK_α_ radiation (λ = 0.71073 Å). The φ- and ω-scan techniques were employed to measure intensities. Absorption corrections were applied with the use of the SADABS program. The crystal structures were solved using the SHELXT [31] and were refined using SHELXL [32] programs with OLEX2 GUI [33]. Atomic displacement parameters for non-hydrogen atoms were refined anisotropically except for C atoms in **2**. The structures were deposited to the Cambridge Crystallographic Data Centre (CCDC) as a supplementary publication No. 2131021–2131026.

## 3. Results and Discussion

### 3.1. Synthesis and Characterization

**Scheme 2:**
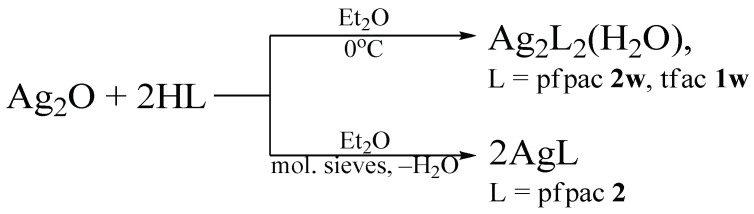
The synthesis routes to obtain Ag(I) fluorinated β-diketonates.

The syntheses of both Ag(I) fluorinated β-diketonates were performed by a convenient acid–base reaction between silver oxide and the corresponded β-diketone [6,15,16,17,19]. The reactions were carried out at ambient conditions using diethyl ether as a solvent. The generated water (half-equivalent, Scheme 2) tends to be incorporated into the resulting compound. The aqua-β-diketonates of Ag_2_(L)_2_(H_2_O) stoichiometry was previously formed both for symmetric and asymmetric perfluorinated ligands: R^1^ = R^2^ = CF_3_ [19], R^1^ = C_3_F_7_, R^2^ = C(CH_3_)_3_ [6]. However, anhydrous compounds were straightly obtained in some cases: R^1^ = CF_3_, R^2^ = C(CH_3_)_3_ [16] R^2^ = C(OCH_3_)(CH_3_)_2_ [17].

In this work, we received aqua-β-diketonates **1w** and **2w**. The tendency to form anhydrous crystals upon slow crystallization at low temperatures was previously noted for [Ag(tfac)]_∞_ **1** [15]. We confirmed this observation for both compounds, obtaining **1** and **2** crystals by another crystal growth method. Moreover, for the new pfpac-ligand, we have implemented an alternative strategy for the direct synthesis of the anhydrous coordination polymer **2** (Scheme 2). The idea was to capture the generated water, for which we used molecular sieves. The Schlenk technique was applied to prevent the capture of water from the environment. In this synthetic method, it was important that the product did not form as a precipitate, but was completely dissolved in the solvent used. This was easily achieved, due to observed significant increase in the solubility of the Ag(I) β-diketonate compound in ether with an increase in the fluorinated substituent length (evaluated up to 850 mg **2w**/100 mL Et_2_O).

The IR spectra of the obtained Ag(I) β-diketonates contain several characteristic series of bands. Stretching vibrations of C–H bonds (3000–2800 cm^−1^) appeared for all compounds, while vibrations of the O–H bonds (3500–3200 cm^−1^) of water can be seen only in spectra of **1w** and **2w**. This was confirmed by the recoding of spectra in fluorinated oil. The wavenumber region of 1650–1100 cm^−1^ contains several strong and medium peaks of following vibrations: stretching C=O (~1640, 1553–1518, ~1465, 1360 cm^−1^), stretching C–F (~1190 cm^−1^), and deformation CF_3_ (1130–1120 cm^−1^). Frequencies of some weaker vibrations of C=C and deformation CH_3_ are mostly superimposed with C=O and C–F peaks. It should be noted that peaks of stretching vibration of C–CF_3_ bonds for pfpac-based compounds **2w** and **2a** are strongly shifted towards higher frequencies when compared to tfac-complexes **1**, **1w**, and **1a** (~1330 cm^−1^ versus ~1280 cm^−1^), which can be associated with a change of substituent in thr β-diketonate ligand. Peaks in the range of 1030–900 cm^−1^ are associated with rotational –CH_3_ vibrations (1030–1020 cm^−1^, ~990 cm^−1^) and stretching C–C vibrations (935–920 cm^−1^). It is interesting that the peak at ~1030–1020 cm^−1^ is much more intensive for **2w** and **2a** than for **1**, **1w**, and **1a**, which can also be associated with the influence of the terminal substituent in the ligand. The region of 850–500 cm^−1^ contains some medium and weak peaks of stretching and deformation C–C vibrations (~820 cm^−1^), π-system of β-diketonate vibrations (810–800 cm^−1^), and vibrations of chelate metallocycle (~760 cm^−1^), as well as Ag–O (~760 cm^−1^, 620–600 cm^−1^) and Ag–C (~725 cm^−1^, 570–550 cm^−1^, 530–516 cm^−1^). Some of these peaks are superimposed. Several typical IR spectra are given in Supplementary Materials (Figure S1). The set of bands in the IR spectra of compound **1** fully corresponds to that previously described in the work [15], which was used for detailed assignment.

According to NMR data, there was no free β-diketonate ligand in the samples, which was confirmed by the absence of the OH-group resonance in the ligands enol-form (resonance with chemical shift of more than 10 ppm). Coordination of the β-diketonate ligand to the silver leads to a slight shift of the C_α_–H group resonance to the high-field region.

All the silver β-diketonates studied (**1**, **2**, **1w**, **2w**) are slowly soluble in water, but quickly soluble in polar O-donor solvents (alcohols, THF). However, the time-dependent stability of these solutions requires further study, since precipitation of silver or its oxide was observed upon exposure to light or heating. They also dissolved in N-donor (CH_3_CN) and aromatic (toluene) solvents, and the corresponding adducts Ag_2_(L)_2_(Q) were formed upon evaporation. The stoichiometry of these products was confirmed by ^1^H-NMR. This composition seems to be typical for the adducts of Ag(I) β-diketonates, since it was noted for various solvents and anionic ligands [16,20,21]. It was retained when the adduct crystals **1t**, **2a,** and **2t** were grown from appropriate solvents. The exception was the acetonitrile adduct of Ag(tfac), whose crystals **1a** correspond to one-equivalent stoichiometry.

It should be noted that the formation of the adducts with CH_3_CN were also demonstrated by IR through the appearance of a characteristic band at 2310 and 2280 cm^−1^, corresponding to C≡N stretching vibrations (Figure S1, Supplementary Materials). At the same time, the bands of water vibrations disappeared. Both acetonitrile adducts Ag_2_(L)_2_(CH_3_CN) are storage stable, while the toluene adducts are rapidly converted to the corresponding aqua-β-diketonates **1w** and **2w**. According to NMR, in the studied solutions, both toluene and acetonitrile most likely leave the metal coordination sphere, which is confirmed by the values of their chemical shifts, corresponding to free solvents in DMSO.

### 3.2. Structural Features of Ag(I) Fluorinated β-Diketonates and Their Adducts

**Figure 1:**
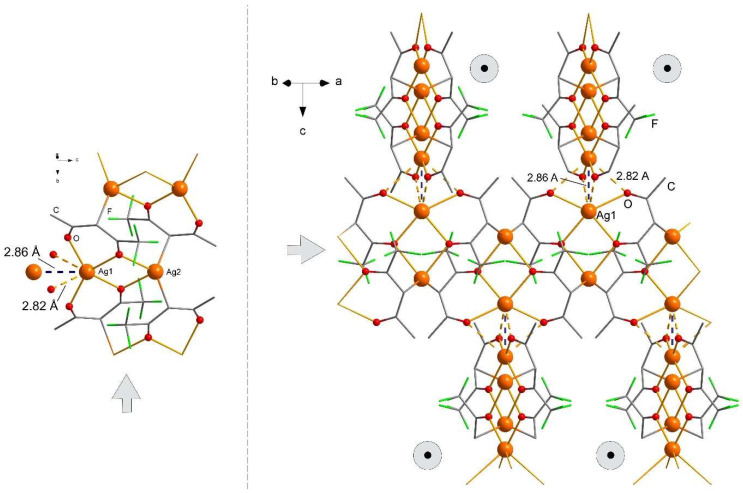
Fragments of the structure of compound [Ag (tfac)]_∞_ **1** in two projections. C and F atoms are shown in a sticks model, H atoms are omitted. Dashed lines indicate Ag···O and Ag···Ag contacts longer than 2.60 Å. Centered circles indicate perpendicular to the figure direction of chain propagation, arrows indicate a vertical or horizontal direction. Disorder of the CF_3_ group is not shown. Atoms’ coloring scheme: orange—Ag, red—O, gray—C, green—F.

**Figure 2:**
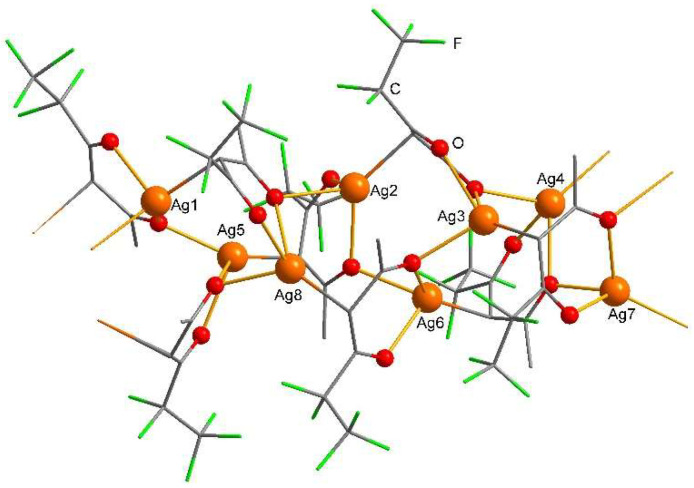
A fragment of the structure of compound [Ag (pfpac)]_∞_ **2**. C and F atoms are shown in a sticks model, H atoms are omitted. Atoms’ coloring scheme: orange—Ag, red—O, gray—C, green—F.

**Figure 3:**
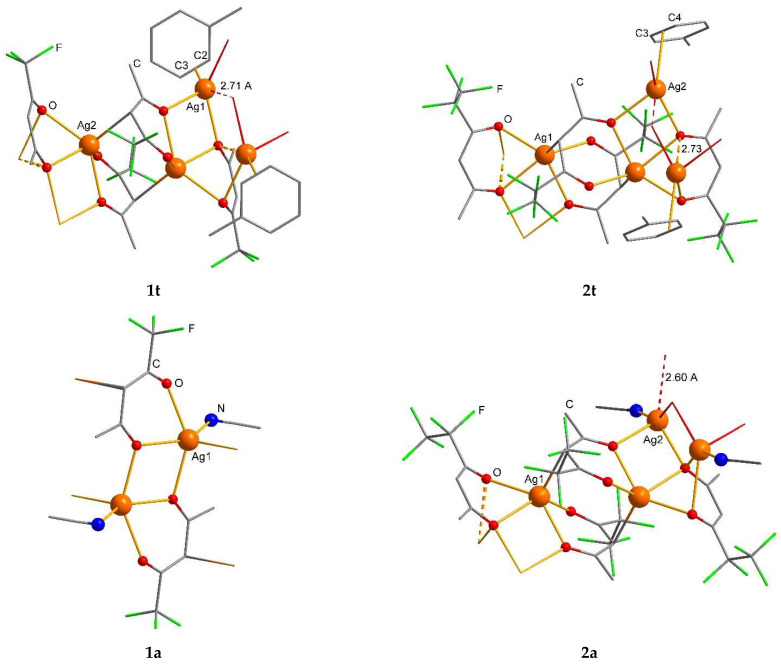
A fragment of the structure of the compounds **1t**, **2t**, **1a** and **2a**. C and F atoms are shown in a sticks model, H atoms are omitted. Dashed lines indicate Ag···O contacts longer than 2.60 Å. Atoms’ coloring scheme: orange—Ag, red—O, gray—C, green—F, blue—N.

According to single-crystal XRD data, the compounds under study (**1**, **2**, **1a**, **2a**, **1t**, **2t**) reveal chain-like structures of different spatial geometry that varies depending on the diketonate (tfac vs. pfpac) and the presence of toluene or acetonitrile ligands. The coordination number (CN) of Ag atoms equals four in the case of non-solvent-containing compounds **1** and **2**, while it equals five for the adducts. Similar to related Ag coordination polymers [17], the coordination polyhedron is highly distorted. For most of the central atoms of **1** and **2**, it can be best described as a vacant trigonal bipyramid (*C*_3*v*_) according to the continuous shape measure analysis (Table S2, Supplementary Materials) [34]. For the Ag atoms of **1a**, **2a**, **1t,** and **2t**, the polyhedron is best described as a spherical square pyramid (*C*_4*v*_) (Table S3, Supplementary Materials). Selected parameters of the silver coordination environment and chelate metallocycles are listed in Table 1 for the solvent-free coordination polymers (**1**, **2**) and in Table 2 for the adducts (**1a**, **2a**, **1t**, **2t**). The fragments of the structures are presented in Figure 1, Figure 2 and Figure 3.

The β-diketonate ligands in compounds **1**, **2**, and **1a** have a µ_3_-κ^1^:κ^1^:κ^2^ chelate-bridging function via one C and two O atoms. Compounds **1t**, **2t,** and **2a** comprise two types of the β-diketonates: one of them is the same as in the former cases, while another is µ_2_-κ^1^:κ^2^ chelate-bridging only via O atoms. The toluene ligands coordinate in η^2^ manner; however, in **1t**, it binds via C^2^ and C^3^ atoms, while in **2t** via C^3^ and C^4^ atoms. Such contacts at a relatively large distance (Table 2) lead to a low stability of these adducts (Par. 3.1). The difference in the coordination mode is likely caused by steric reasons. Note that the Ag–N (acetonitrile) bond is noticeably shorter in **2a** when compared to **1a**, which is probably also a consequence of steric effects and the presence of the stronger electron-withdrawing group (C_2_F_5_ vs. CF_3_).

The bond lengths Ag–O vary over a wide range of 2.26 (1)–2.732 (3) Å, generally being shorter for the oxygens belonging to the chelate mode (Table 1 and Table 2). In the adduct structure of one-equivalent stoichiometry **1a** (Figure 3), the chain appears less tense, and is characterized by the largest minimum distances between the silver atoms (Table 2). The range of Ag–O bond lengths here corresponds to solvent-free Ag (I) β-diketonates (Table 1). For the adducts of half-stoichiometry **1t**, **2t,** and **2a**, there are elongated Ag–O distances exhibited by the Ag atom that connected with a solvent molecule (Table 2). In the case of toluene adducts **1t** and **2t**, these distances are realized with µ_3_-O of β-diketonate-ligand (~2.72 Å), while for the acetonitrile adduct **2a** the distance is noticeably shorter (~2.60 Å) and realized with µ_2_-O.

It should be noted that the structures of **2** and all the adducts belong to the chain polymeric motif. In compound **1**, each chain is connected via short contacts Ag···Ag of 2.82 Å with the neighboring ones, spreading in the perpendicular direction (Figure 1). As a result, compound **1** features a 3D coordination framework.

To construe the observed structural diversity, we analyzed in the same manner the structures presented in this work and the earlier published toluene and acetonitrile adducts:

1. [Ag_2_ (hfac)_2_ (C_7_H_8_)] **3t** [20] (hfac = 1,1,1,5,5,5-hexafluoro-3,5-pentanedionate, R^1^ = R^2^ = CF_3_);
2. [Ag_2_ (btfac)_2_ (CH_3_CN)] **4a** [21] (btfac = 4,4,4-trifluoro-1-phenyl-1,3-butanedionate, R^1^ = CF_3_, R^2^ = C_6_H_5_);
3. [Ag_2_ (nphtfac)_2_ (CH_3_CN)] **5a** [21] (nphtfac = 4,4,4-trifluoro-1-(2-naphthyl)-1,3-butanedione, R^1^ = CF_3_, R^2^ = C_10_H_7_).

We distinguished several types of secondary building units (SBUs) in these structures.

**Figure 4:**
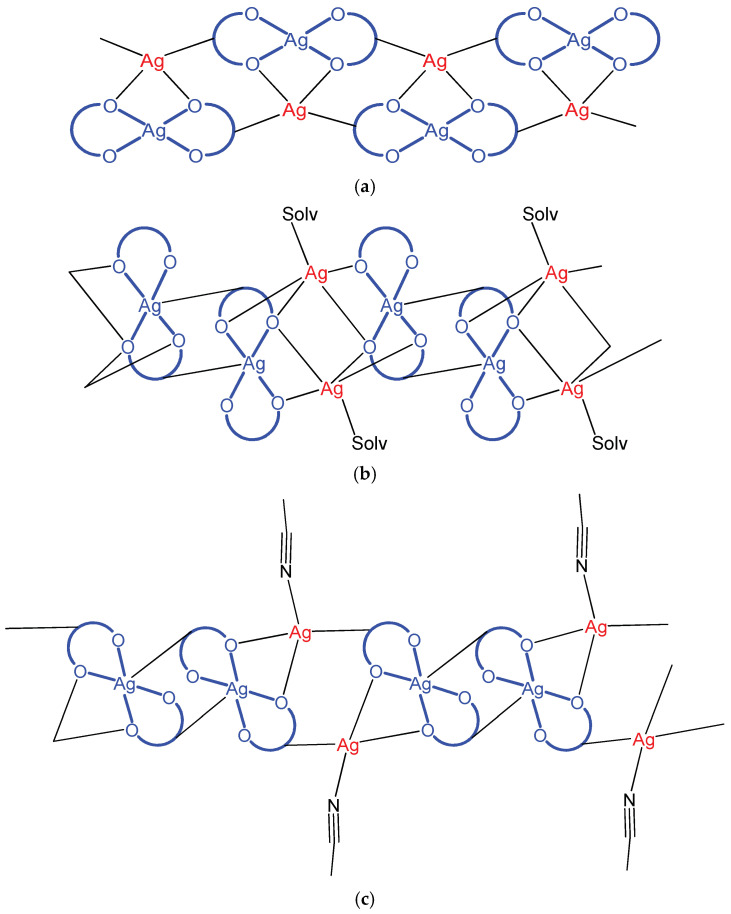
Schematic representation of (**a**) [Ag (tfac)]_∞_ **1**; (**b**) [Ag_2_ (pfpac)_2_ (CH_3_CN)] **2a** and [Ag_2_ (L)_2_ (C_7_H_8_)] (L = tfac **1t**, pfpac **2t**); (**c**) [Ag_2_ (L)_2_ (CH_3_CN)] (L = btfac **4a**, nphtfac **5a**). The substituents in β-ligands are omitted for clarity. {Ag (β-dik)_2_} SBUs are marked blue.

The first SBU type is the {Ag (β-dik)_2_} block with two chelating anions. This type was revealed in the structures **1**, two semi-equivalent toluene adducts (**1t** and **2t**), and all the semi-equivalent acetonitrile adducts (**2a**, **4a**, **5a**), and is connected in chains via Ag atoms (Figure 4). In the structure of solvent-free coordination polymers, [Ag (tfac)]_∞_ **1**, the {Ag (β-dik)_2_} SBUs are separated from each other and alternate with Ag atoms coordinating two O and two C atoms (Figure 4a). In all the solvent-containing structures, the {Ag (β-dik)_2_} SBUs are pairwise connected via Ag–Cα bonds. However, Ag in {Ag (solvent)} SBUs coordinates either four O atoms (two oxygens from one ligand of the first {Ag (β-dik)_2_} and two oxygens from two ligands of the second {Ag (β-dik)_2_}) in the case of **2a**, **1t**, **2t** (Figure 4b), or two O and one C atom in the case of **4a** and **5a** (Figure 4c). The different motifs of the chains for the adducts likely arise from different substituents in the β-diketonate backbone: the latter two structures comprise bulky aryl groups, while our structures comprise small CH_3_ groups.

**Figure 5:**
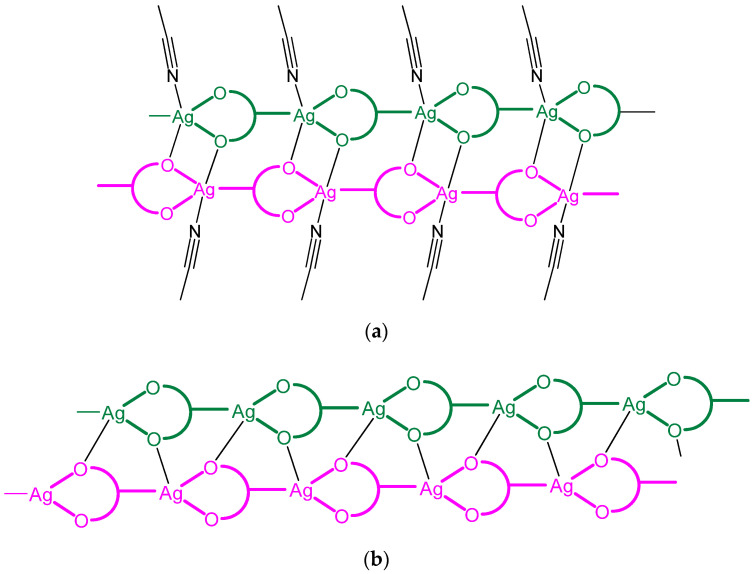
Schematic representation of (**a**) [Ag (tfac) (CH_3_CN)] **1a**; (**b**) [Ag (pfpac)]_∞_ **2**. The substituents in β-ligands are omitted for clarity. {Ag (β-dik)} SBUs are marked green and pink.

**Figure 6:**
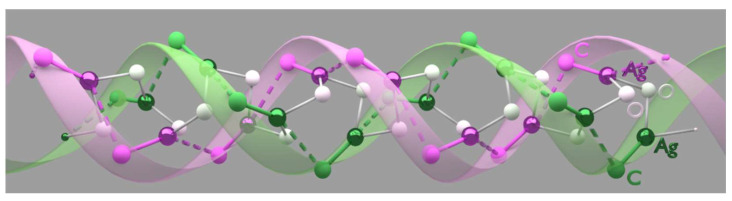
Schematic representation of [Ag (pfpac)]_∞_ **2** stranded structure. Only Ag and bridging O and C atoms are shown. The positions of the ligands are depicted as dashed lines, translucent ribbons show the twist of the chains.

The second SBU type is the {Ag (β-dik)} block with one chelating anion that connects with each other in chains via Ag–C_α_ bonds (Figure 5). These SBUs are presented in [Ag (pfpac)]_∞_ **2** and [Ag (tfac) (CH_3_CN)] **1a**. In both structures, each chain connects with the neighboring one via Ag–O bonds; however, in the adduct **1a**, the two bonds are formed within the pair of {Ag (β-dik)} SBUs (Figure 5a), while in solvent-free coordination polymer **2**, each {Ag (β-dik)} forms Ag–O bonds with two different SBUs (Figure 5b). Interestingly, the latter structure manifests itself as a double stranded one, akin to a DNA strand (Figure 6).

**Figure 7:**
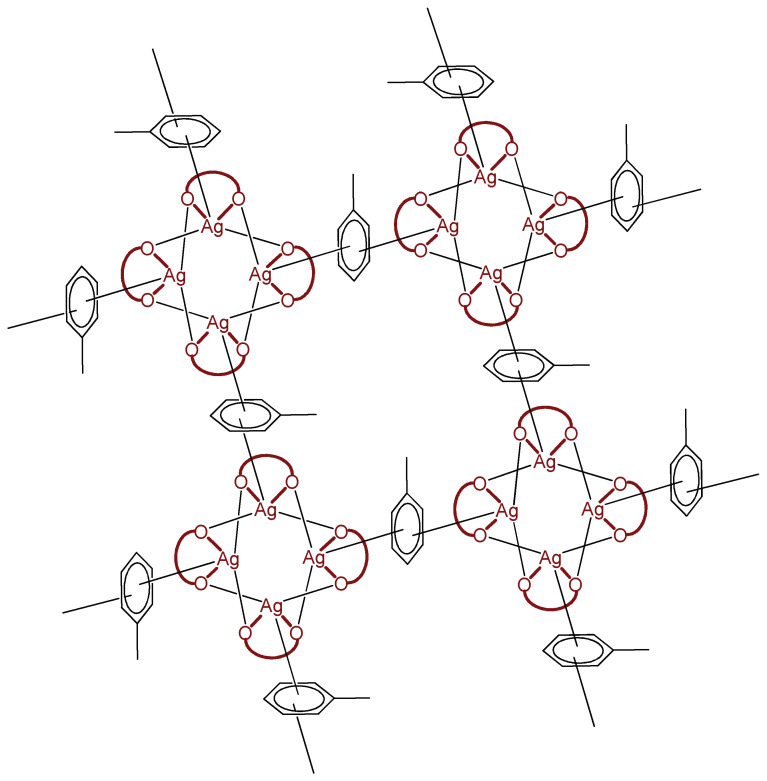
Schematic representation of [Ag_2_ (hfac)_2_ (C_7_H_8_)] **3t**. {Ag (β-dik)} SBUs are marked wine.

Finally, it is interesting to note that, despite the same stoichiometry, the structure of the toluene adduct **4t** with the hfac-ligand is fundamentally different from the adducts **1t** and **2t** obtained in this work. The coordination polymer **4t** exhibits not a chain, but a layered motif, where the {Ag (β-dik)} SBUs are connected via Ag–O bonds into tetranuclear fragments, which are further connected by bridging toluene ligands (Figure 7). In contrast to the compounds discussed above, the structure **4t** features no Ag–Cα bonds with the β-diketonate.

### 3.3. Thermal Analysis

**Figure 8:**
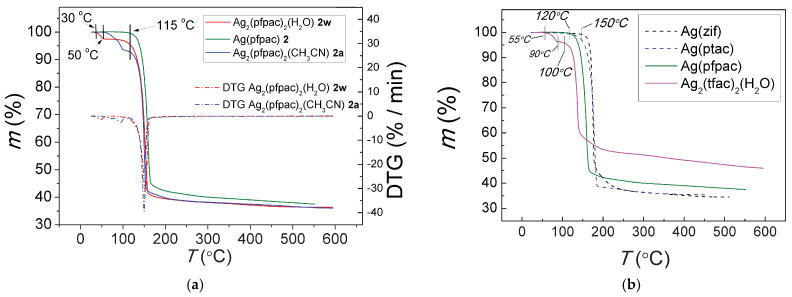
Thermal analysis data for (**a**) [Ag (pfpac)]_∞_ **2** and its water **2w** and acetonitrile **2a** adducts (**b**) a row of Ag (I) compounds with fluorinated β-diketonates R^1^C (O)C_α_HC (O)R^2^: R^1^ = C_2_F_5_, R^2^ = CH_3_ (pfpac), R^1^ = CF_3_, R^2^ = CH_3_ (tfac), C (CH_3_)_3_ (ptac), C (OCH_3_) (CH_3_)_2_ (zif).

Thermal properties of the compounds were studied in an inter-atmosphere (helium flow). For a new family of Ag (I) pfpac-compounds we compared the behavior of pristine β-diketonate **2** with its water **2w** and acetonitrile **2a** adducts (Figure 8a).

The mass loss of the adducts starts from the solvent elimination: for **2w**: calcd. ω (½H_2_O) = 2.9%, exp. the first mass loss step = 2.6%; for **2a**: calcd. ω (½CH_3_CN) = 6.2%, exp. the first mass loss step = 6.6%. It should be noted that water is lost at 30–60 °C, while acetonitrile is lost at higher temperatures (50–90 °C). This may indicate a weaker binding of water molecules. Moreover, according to differential TG curves (DTG), water eliminates at one step, while CH_3_CN leaves the coordination sphere in two steps (Figure 8a). According to c-DTA, solvent removal is endothermic.

The solvent-free coordination polymer **2** is stable up to roughly 115 °C (for all samples), and the major mass loss proceeds in one step and is completed before 200 °C. Then, a slow small mass loss occurs, which can be attributed to the annealing of the carbon products of the compounds’ decomposition. XRPD shows a single crystalline decomposition product, namely, metallic silver (PDF 010–89 3722 [35]). However, the calculations of the mass loss curves allow the suggesting of the presence of an amorphous component: for **2w** calcd. ω (Ag) = 33.7%, exp. ω (residue) = 36.4%; for **2a** calcd. ω (Ag) = 32.5%, exp. ω (residue) = 36.0%; for **2** calc. ω (Ag) = 34.7%, exp. ω (residue) = 37.5%). This component is likely an unannealed amorphous carbon.

Within a family of tfac-based compounds, aqua-β-diketonate **1w** was studied (Figure 8b). Similar to **2w**, the first mass loss step corresponds to endothermic water elimination: calcd. ω (½H_2_O) = 3.3%, exp. mass loss value = 3.7%). However, this process occurs at higher temperatures (55–90 °C). Apparently, in adduct **1w**, water is connected by stronger interactions than in **2w**, despite the presence of a stronger electroacceptor substituent in **2w** (C_2_F_5_ versus CF_3_). This may indicate completely different structures of the considered aqueous adducts. The decomposition of solvent-free coordination polymer **1** starts at 100 °C.

The thermal behavior of the Ag (I) β-diketonates considered here was compared with the data obtained under similar conditions for the related solvent-free fluorinated [Ag (R^1^C (O)C_α_HC (O)R^2^]_∞_ compounds containing a bulky non-fluorinated substituent: R^1^ = CF_3_, R^2^ = C (CH_3_)_3_ (ptac), C (OCH_3_) (CH_3_)_2_ (zif) (Figure 8b). All these complexes were decomposed to metallic silver (XRPD). Herewith, Ag (tfac) **1,** which alone contains argentophilic interactions in the structure, is the least terminally stable, while Ag (zis) where the silver coordination is supplemented by an Ag–O_CH3_ bond, is the most stable. The other two β-diketonates are comparable in thermal stability.

## 4. Conclusions

Silver (I) β-diketonate complexes exhibit great potential for structural design and the formation of various building blocks, both due to the various modes of ligand coordination (by oxygen atoms and/or methine carbon) and via the inclusion of solvents. Herein, we have investigated the effect of a fluorinated substituent (CF_3_/C_2_F_5_) and N-donor (CH_3_CN) and aromatic (toluene) solvents on the structural and thermal properties of this class of the silver compounds. The results obtained were analyzed in comparison with a series of related, fluorinated Ag (I) β-diketonates and their acetonitrile and toluene adducts. Thus, we have identified characteristic secondary building units and several new types of structural organization of these coordination polymers.

In particular, for fluorinated Ag (I) β-diketonates, the coordination polymer with a small-size methyl substituent (tfac) possesses a 3D framework structure based on argentophilic interactions, and therefore is the least thermally stable. An increase of bulkiness in both fluorinated (CF_3_ → C_2_F_5_) and non-fluorinated (CH_3_ → C(CH_3_)_3_/C(OCH_3_) (CH_3_)_2_/C_6_H_5_) substituents leads to the expected decrease in the structural dimension to 2D (double chains). The mutual repulsion of large-size fluorinated substituents (C_2_F_5_) leads to structural reorganization and helical twisting of two chains.

For Ag (I) β-diketonate adducts, the stoichiometry is not the main structure-determining parameter. In fact, three motifs of structural organization were found for [Ag_2_ (L)_2_ (Q)]_∞_ polymers (Q = CH_3_CN, toluene). At the same time, these adducts with different β-diketonate-ligand and solvents can form the same structure motif. This includes the chain structures of both CH_3_CN and toluene adducts with asymmetric fluorinated β-diketonates (R^1^ = CH_3_, R^2^ = CF_3_/C_2_F_5_). The introduction of an aromatic non-fluorinated substituent into the anion leaves the main structure motif (1D, double chains), changing the chain organization. However, the latter was shown only for acetonitrile adducts. Finally, the presence of two CF_3_-groups in the β-diketonate-anion allows for the bridging function of toluene in the adduct structure. Thus, the combination of solvent and β-diketonate is important here, but more structural data is needed to understand the general trends. However, the adducts with acetonitrile are evidently more stable when compared to that with toluene owing due to better binding.

The obtained compounds broaden a family of silver (I) coordination polymers and might be employed to obtain Ag-containing functional materials. The exploration of their biological properties can also be a subject of further research.

## Figures and Tables

**Table 1:** Selected distances and angles in the structures [Ag(tfac)]_∞_ **1** and [Ag(pfpac)]_∞_ **2**.

Coordination Polymer	[Ag (tfac)]_∞_	[Ag (pfpac)]_∞_
Identification code	**1**	**2**
Distance, Å		
Ag–O_β-dik_ (chelate, non-bridging)	2.328 (6), 2.374 (6)	2.37 (1)–2.46 (2)
Ag–O_β-dik_ (chelate, bridging)	2.374 (6)	2.37 (1)–2.43 (1)
Ag–O_β-dik_ (non-chelate, bridging)	2.466 (6)	2.26 (1)–2.37 (1)
Ag–C_α_	2.336 (8)	2.25 (2)–2.32 (2)
Ag···Ag (min., in the chain)	3.666 (2)	3.43 (1)–4.25 (1)
Ag···Ag (min., between the chains)	2.861 (2)	7.80(1)
Angle, °		
O–Ag–O_β-dik_ (chelate)	77.0 (3)	71.9 (5)–76.2 (5)
Deformation of chelate metallocycle AgO_2_C_3_: angle between the planes, °		
(Ag, O, O_β_) & (O, C, C_β_, O_β_)	162.2	156.4–159.8
(C, C_α_, C_β_) & (O, C, C_β_, O_β_)	170.1	168.5–172.2

**Table 2:** Selected distances and angles in the structures of the adducts **1a**, **2a**, **1t,** and **2t**. Elongated Ag–O bonds are underlined. If noted, the additional designation of silver atoms means: Ag(NS) = non-solvent-connected Ag atom, Ag(S) = solvent-connected Ag atom.

General Formula	[Ag_2_ (L)_2_ (Q)]_∞_			
L	tfac		pfpac	
Q	CH_3_CN, X = N	C_7_H_8_, X = C	CH_3_CN, X = N	C_7_H_8_, X = C
Identification Code	1a	1t	2a	2t
Distance, Å				
Ag (S)–X	–	2.650 (2), 2.548 (2)	2.180 (3)	2.481 (5)
Ag (NS)–O_β-dik_ (chelate, non-bridging)	–	2.344 (2)	2.370 (2)	2.346 (3)
Ag (S)–O_β-dik_ (chelate, non-bridging)	2.395 (2)	–	–	–
Ag (NS)–O_β-dik_ (chelate, bridging)	–	2.412 (2)–2.455 (2)	2.358 (2)–2.472 (2)	2.365 (3)–2.515 (4)
Ag (S)–O_β-dik_ (chelate, bridging)	2.439 (2)	2.388 (2), 2.714 (2)	2.474 (2), 2.596 (2)	2.484 (4), 2.732 (3)
Ag (S)–O_β-dik_ (non-chelate, bridging)	2.459 (2)	2.397 (2), 2.404 (2)	2.326 (2), 2.488 (2)	2.292 (3), 2.432 (4)
Ag (NS)–C_α_	–	2.369 (2)	2.390 (3)	2.487 (5)
Ag (S)–C_α_	2.468 (2)	–	–	–
Ag···Ag (min., in the chain)	3.820 (2)	3.1235 (4)	3.2379 (3)	3.1087 (6)
Angle, °				
O–Ag–O_β-dik_ (chelate)	75.40 (6)	67.53 (5)–76.67 (6)	66.79 (7)–75.82 (8)	69.7 (1)–77.1 (1)
Ag···Ag···Ag (in the chain)	71.9, 108.1	69.8, 110.2	64.0, 116.0	72.4, 107.6
Deformation of chelate metallocycle AgO_2_C_3_: angle between the planes, °				
(Ag, O, O_β_) & (O, C, C_β_, O_β_)	160.8	130.3–147.3	132.0, 148.4	123.4–153.4
(C, C_α_, C_β_) & (O, C, C_β_, O_β_)	173.2	169.4, 178.3	172.6, 179.0	175.4, 176.3

## Data Availability

The data presented in this study are available on request from the corresponding author. The CIF files containing full crystallographic data for **1**, **2**, **1a**, **2a**, **1t**, **2t** (CCDC 2131021–2131026) can be obtained free of charge via http://www.ccdc.cam.ac.uk/conts/retrieving.html (accessed on 27 December 2021) (or from the CCDC, 12 Union Road, Cambridge CB2 1EZ, UK; Fax: +44-1223-336033; E-mail: deposit@ccdc.cam.ac.uk).

## Supplementary Materials

The following supporting information can be downloaded at. Figure S1: Typical IR spectra of the samples; Table S1: Crystal data and structure refinement for the compounds; Table S2: Geometry analysis of the complexes showing coordination number of Ag of 4 by SHAPE software; Table S3: Geometry analysis of the complexes showing coordination number of Ag of 5 by SHAPE software.

## Sample Availability

Samples of the compounds **1**, **2**, **1a**, **2a**, **1t,** and **2t** are available from the authors.
