# Supplementary material for: Structural Diversity of Silver Fluorinated β-Diketonates: Effect of the Terminal Substituent and Solvent
Source: Molecules. 2022 Jan 20;27(3):677. doi: 10.3390/molecules27030677 (PMC8839988; doi:10.3390/molecules27030677)
Supplement: Supplementary file 1 [file molecules-27-00677-s001.zip › molecules-1557024-supplementary.pdf]

Supplementary Materials

Structural Diversity of Silver Fluorinated β-Diketonates: Effect of the Terminal Substituent and Solvent

Evgeniia S. Vikulova [1]*, *Taisiya S. Sukhikh* [1], *Sergey A. Gulyaev* [1,2], *Igor Yu. Ilyin* [1] *and Natalia B. Morozova* [1]

[1] Nikolaev Institute of Inorganic Chemistry, Siberian Branch of Russian Academy of Sciences, 3 Acad. Lavrentiev Ave., 630090, Novosibirsk, Russia; lazorevka@mail.ru (E.S.V.), sukhikh@niic.nsc.ru (T.S.S.), ilyin@niic.nsc.ru (I.Y.I.), mor@niic.nsc.ru (N.B.M.)

[2] Novosibirsk State University, 2 Pirogova Str., 630090, Novosibirsk, Russia; s.gulyaev@g.nsu.ru (S.A.G.)

* Correspondence: lazorevka@mail.ru; Tel.: +7 383 3309556

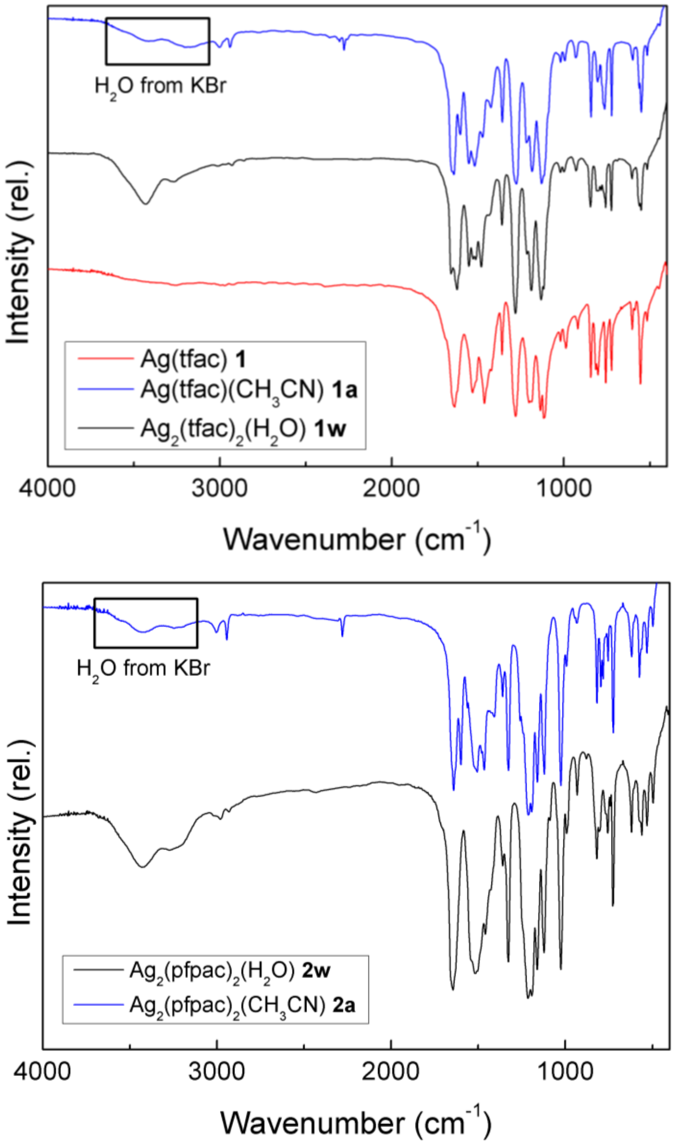

Figure S1. Typical IR spectra of the samples.

Table S1. Crystal data and structure refinement for the compounds.

Identification code	**1**	**1a**	**1t**	**2**	**2a**	**2t**
Empirical formula	$C_5H_4AgF_3O_2$	$C_{14}H_{14}Ag_2F_6N_2O_4$	$C_{17}H_{16}Ag_2F_6O_4$	$C_6H_4AgF_5O_2$	$C_{14}H_{11}Ag_2F_{10}NO_4$	$C_{19}H_{16}Ag_2F_{10}O_4$
Formula weight	260.95	604.01	614.04	310.96	662.98	714.06
Temperature/K	150(2)	150(2)	150(2)	150(2)	150(2)	150(2)
Space group	*Fddd*	*P*–1	*P*–1	$P2_1/n$	*P*–1	*P*–1
a/Å	11.3887(9)	5.0839(10)	8.3396(9)	14.531(4)	8.6581(3)	8.6850(6)
b/Å	15.0409(11)	8.1716(17)	10.7518(12)	24.835(7)	10.9844(3)	11.7496(7)
c/Å	31.601(2)	11.737(2)	11.6428(12)	19.833(5)	12.5306(6)	12.6738(9)
α/°	90	88.158(7)	105.470(4)	90	64.3360(10)	62.928(3)
β/°	90	78.284(6)	102.392(3)	100.861(8)	74.069(2)	80.634(4)
γ/°	90	75.566(7)	90.890(4)	90	82.9590(10)	72.814(3)
Volume/$Å^3$	5413.1(7)	462.29(16)	979.76(18)	7029(3)	1032.88(7)	1099.58(13)
Z	32	1	2	32	2	2
$\rho_{calc}g/cm^3$	2.562	2.170	2.081	2.351	2.132	2.157
μ/mm^{-1}	2.979	2.199	2.075	2.346	2.005	1.890
F(000)	3968.0	292.0	596.0	4736.0	636.0	692.0
Crystal size/mm^3	0.1 × 0.06 × 0.05	0.11 × 0.03 × 0.03	0.22 × 0.08 × 0.07	0.51 × 0.03 × 0.03	0.3 × 0.17 × 0.12	0.35 × 0.2 × 0.05
Radiation	MoKα (λ = 0.71073)	MoKα (λ = 0.71073)	MoKα (λ = 0.71073)	MoKα (λ = 0.71073)	MoKα (λ = 0.71073)	MoKα (λ = 0.71073)
2Θ range for data collection/°	5.156 to 52.77	5.148 to 61.066	4.616 to 63.194	3.204 to 41.632	4.114 to 51.504	4.912 to 51.788
Index ranges	-14 ≤ h ≤ 14, -18 ≤ k ≤ 18, -39 ≤ l ≤ 36	-7 ≤ h ≤ 7, -11 ≤ k ≤ 11, -16 ≤ l ≤ 16	-12 ≤ h ≤ 12, -15 ≤ k ≤ 15, -17 ≤ l ≤ 17	-14 ≤ h ≤ 14, -24 ≤ k ≤ 24, -19 ≤ l ≤ 19	-10 ≤ h ≤ 10, -13 ≤ k ≤ 10, -15 ≤ l ≤ 15	-10 ≤ h ≤ 9, -14 ≤ k ≤ 14, -15 ≤ l ≤ 15
Reflections collected	18733	8262	18647	50735	9845	9659
Independent reflections	1383 [R_{int} = 0.0276, R_{sigma} = 0.0121]	2819 [R_{int} = 0.0263, R_{sigma} = 0.0306]	6499 [R_{int} = 0.0479, R_{sigma} = 0.0472]	7369 [R_{int} = 0.1574, R_{sigma} = 0.1006]	3856 [R_{int} = 0.0239, R_{sigma} = 0.0264]	4209 [R_{int} = 0.0595, R_{sigma} = 0.0742]
Data/restraints/parameters	1383/0/131	2819/0/129	6499/0/265	7369/42/777	3856/0/283	4209/0/319
Goodness-of-fit on F^2	1.374	1.062	1.079	1.016	1.030	1.015
Final R indexes [I>=2σ (I)]	R_1 = 0.0524, wR_2 = 0.1154	R_1 = 0.0263, wR_2 = 0.0616	R_1 = 0.0364, wR_2 = 0.0973	R_1 = 0.0773, wR_2 = 0.1817	R_1 = 0.0257, wR_2 = 0.0617	R_1 = 0.0488, wR_2 = 0.1188
Final R indexes [all data]	R_1 = 0.0548, wR_2 = 0.1163	R_1 = 0.0303, wR_2 = 0.0631	R_1 = 0.0392, wR_2 = 0.1012	R_1 = 0.1265, wR_2 = 0.2084	R_1 = 0.0308, wR_2 = 0.0652	R_1 = 0.0602, wR_2 = 0.1276
Largest diff. peak/hole / e $Å^{-3}$	0.82/-1.17	0.66/-1.25	1.71/-1.81	4.07/-1.24	0.61/-0.56	1.51/-1.61

Table S2. Geometry analysis of the complexes showing coordination number of Ag of 4 by SHAPE software. The best description of the polyhedral is marked green.

Complex	Central atom	Square (D_{4h})	Tetrahedron (*Td*)	Seesaw (C_{2v})	Vacant trigonal bipyramid (C_{3v})
**1**	Ag1	6.427	20.309	12.383	19.861
	Ag2	30.85	3.247	5.072	3.011
**2**	Ag1	32.177	5.235	5.516	3.152
	Ag2	29.228	5.307	6.328	3.614
	Ag3	27.809	5.2	5.126	4.259
	Ag4	31.713	4.535	7.087	3.393
	Ag5	27.493	6.79	7.012	4.649
	Ag6	30.491	5.536	6.288	4.542
	Ag7	33.795	4.009	5.79	2.19
	Ag8	25.71	6.672	5.783	5.553

*SHAPE 2.1 program for the stereochemical analysis of molecular fragments by means of continuous shape measures and associated tools. http://www.ee.ub.edu/]

Table S3. Geometry analysis of the complexes showing coordination number of Ag of 5 by SHAPE software. The best description of the polyhedral is marked green.

Complex	Central atom	Pentagon (D_{5h})	Vacant octahedron (C_{4v})	Trigonal bipyramid (D_{3h})	Spherical square pyramid (C_{4v})	Johnson trigonal bipyramid J12 (D_{3h})
**1a**	Ag1	25.267	3.83	6.219	1.516	9.412
**1t**	Ag1*	24.158	5.217	8.168	5.063	8.361
	Ag2	26.738	5.951	4.876	2.125	8.496
**2a**	Ag1	26.9	5.586	5.67	2.011	9.683
	Ag2	23.743	7.34	7.806	7.28	10.632
**2t**	Ag1	21.546	6.277	6.979	3.637	10.697
	Ag2	23.982	8.427	9.694	8.448	10.142
	Ag2*	26.606	9.657	12.134	8.683	12.651

* one of the vertices is a dummy atom lying in the middle between two C atoms of the toluene ligand.
